# The Role of *nmcR*, *ampR*, and *ampD* in the Regulation of the Class A Carbapenemase NmcA in *Enterobacter ludwigii*

**DOI:** 10.3389/fmicb.2021.794134

**Published:** 2022-01-12

**Authors:** Ryuichi Nakano, Yuki Yamada, Akiyo Nakano, Yuki Suzuki, Kai Saito, Ryuji Sakata, Miho Ogawa, Kazuya Narita, Akio Kuga, Akira Suwabe, Hisakazu Yano

**Affiliations:** ^1^Department of Microbiology and Infectious Diseases, Nara Medical University, Kashihara, Japan; ^2^Division of Central Clinical Laboratory, Iwate Medical University Hospital, Yahaba, Japan; ^3^Department of Bacteriology, BML Inc., Kawagoe, Japan; ^4^Hamamatsu Pharmaceutical Association, Hamamatsu, Japan; ^5^Department of Laboratory Medicine, Iwate Medical University School of Medicine, Yahaba, Japan

**Keywords:** carbapenemase, NmcA, AmpC β-lactamase, *Enterobacter* cloacae complex, induction, regulator genes

## Abstract

Various carbapenemases have been identified in the Enterobacteriaceae. However, the induction and corresponding regulator genes of carbapenemase NmcA has rarely been detected in the *Enterobacter cloacae* complex (ECC). The NmcA-positive isolate ECC NR1491 was first detected in Japan in 2013. It was characterized and its induction system elucidated by evaluating its associated regulator genes *nmcR*, *ampD*, and *ampR*. The isolate was highly resistant to all β-lactams except for third generation cephalosporins (3GC). Whole-genome analysis revealed that *bla*_NmcA_ was located on a novel 29-kb putatively mobile element called EludIMEX-1 inserted into the chromosome. The inducibility of β-lactamase activity by various agents was evaluated. Cefoxitin was confirmed as a strong concentration-independent β-lactamase inducer. In contrast, carbapenems induced β-lactamase in a concentration-dependent manner. All selected 3GC-mutants harboring substitutions on *ampD* (as *ampR* and *nmcR* were unchanged) were highly resistant to 3GC. The *ampD* mutant strain NR3901 presented with a 700 × increase in β-lactamase activity with or without induction. Similar upregulation was also observed for *ampC* and *nmcA*. NR1491 (pKU412) was obtained by transforming the *ampR* mutant (135Asn) clone plasmid whose expression increased by ∼100×. Like NR3901, it was highly resistant to 3GC. Overexpression of *ampC*, rather than *nmcA*, may have accounted for the higher MIC in NR1491. The *ampR* mutant repressed *nmcA* despite induction and it remains unclear how it stimulates *nmcA* transcription via induction. Future experiments should analyze the roles of *nmcR* mutant strains.

## Introduction

The *Enterobacter cloacae* complex (ECC) have become clinically significant opportunistic bacteria and are now common nosocomial pathogens causing pneumonia, urinary tract infections, and septicemia (Davin-Regli and Pages, 2015). Six *Enterobacter* species are assigned to the ECC: *E. cloacae*, *E. asburiae*, *E. hormaechei*, *E. kobei*, *E. ludwigii*, and *E. nimipressuralis* (Mezzatesta et al., 2012).

Multidrug resistance (MDR) has been observed for the last-resort carbapenems and has led to an increased global interest in Enterobacteriaceae in general and carbapenem-resistant ECC, in particular (Annavajhala et al., 2019). ECC are innately resistant to penicillins, first- and second-generation cephalosporins, and cephamycin due to the chromosomally encoded AmpC β-lactamase genes (serine β-lactamase, Ambler class C). AmpC β-lactamase expression is low but inducible in response to β-lactam exposure and is closely linked to a peptidoglycan recycling system, with the β-lactams imipenem, cefoxitin, and clavulanic acid strong *ampC* inducers (Jacoby, 2009). Regulation of AmpC β-lactamase expression is complex and involves AmpR (a transcriptional regulator of the LysR family), AmpD (a cytosolic amidase), and AmpG (a transmembrane permease) (Guerin et al., 2015). AmpR usually represses *ampC* in the absence of β-lactam inducers, whereas mutations at specific sites in AmpR derepresses AmpC synthesis and results in constitutive AmpC β-lactamase overexpression. Asp135Asn AmpR substitution is correlated with substantial increases in β-lactamase activity in several Gram-negative organisms including ECC, *Citrobacter freundii*, and *Pseudomonas aeruginosa* (Kuga et al., 2000; Caille et al., 2014; Nakano et al., 2017). Mutations that inactivate AmpD permanently induce and increase muropeptide concentrations in the cytoplasm and change the conformation of AmpR so that it becomes a transcriptional activator (Kuga et al., 2000). Specifically, AmpR mutations require site-specific substitution to induce AmpC β-lactamase overexpression whereas AmpD mutations need loss-of-function point mutations (missense mutation) or disruption of the protein carboxy terminus, nonsense mutations, frameshifts, and truncations (Schmidtke and Hanson, 2006). Among the ECC clinical isolates, high-level resistance to third generation cephalosporins (3GC) is caused by constitutive *ampC* overexpression mainly from *ampD* mutations and, more rarely, from *ampR* mutations (Kaneko et al., 2005; Guerin et al., 2015).

Carbapenem resistance in ECC is conferred either through constitutive AmpC β-lactamase overexpression combined with defective outer membrane (porin) permeability or via the acquisition of carbapenemase genes (Annavajhala et al., 2019), with the latter scenario being more common. Carbapenemases hydrolyze most β-lactams including carbapenems and are classified as serine β-lactamases (Ambler class A; KPC type and D; OXA-48 type) or metallo-β-lactamases (Ambler class B; IMP type, VIM type, and NDM type) (Diene and Rolain, 2014; Nakano et al., 2014; Ando et al., 2018). The distributions of these enzymes differ with geographical location: the KPC type occurs in the United States, NDM in the Indian subcontinent, and IMP in Japan (Chavda et al., 2016; Aoki et al., 2018; Peirano et al., 2018). Chromosomally encoded carbapenemase NmcA (Ambler class A) has been sporadically detected in ECC (Walther-Rasmussen and Hoiby, 2007).

NmcA was originally detected in the carbapenem-resistant *E. cloacae* strain NOR-1 isolated in France in 1990 (Nordmann et al., 1993). NmcA has occasionally been detected in *E. cloacae*, *E. asburiae*, and *E. ludwigii* from Europe, North America, and South America (Pottumarthy et al., 2003; Radice et al., 2004; Antonelli et al., 2015; Boyd et al., 2017). A recent study revealed that *bla*_NmcA_ is associated with a novel 29-kb putative Xer-dependent integrative mobile element (EludIMEX-1) inserted into the ECC chromosome (Antonelli et al., 2015). This enzyme hydrolyses different β-lactam agents except for 3GC and has particularly high hydrolytic activity against carbapenems (Nordmann et al., 1993; Mariotte-Boyer et al., 1996). The inducibility of NmcA is similar to AmpC β-lactamase (Pottumarthy et al., 2003), where the LysR family transcriptional regulator gene *nmcR* upstream of *bla*_NmcA_ regulates *nmcA* in the same manner as the *ampR*–*ampC* regulatory system does for AmpC. Additionally, AmpD co-regulates *nmcA* (Naas et al., 2001).

Here, we describe the characteristics of an *nmcA*-positive ECC isolate first observed in Japan. We also elucidate the *nmcA* induction system by evaluating *nmcA* expression in *ampD* and *ampR* mutant strains.

## Materials and Methods

### Bacterial Strains and Antimicrobial Susceptibility Testing

The carbapenem-resistant ECC strain NR1491 was isolated from the urine of a patient in a Japanese hospital in 2013. The species was identified as *E. cloacae* by MicroScan WalkAway plus (Beckman Coulter, Inc., Brea, CA, United States). To evaluate the effects of *ampR* mutation on antimicrobial susceptibility and β-lactamase expression, *ampR* clone plasmids were constructed and used to transform NR1491. The *ampR* clone plasmids (pKU411 and pKU412) used in this study were already previously constructed (Kuga et al., 2000). An *in vitro* ceftazidime-resistant mutant strain NR3901 was isolated from NR1491. Characteristics of the bacterial strains and plasmids used in the present study are listed in Table 1.

**TABLE 1:** Bacterial strains and plasmids used in the present study.

Strain or plasmid	Relevant characteristics	Source or references
**Strains**		
*E. ludwigii* NR1491	Clinical isolate from Japan, resistance to carbapenems	This study
pKU411/NR1491	*E. ludwigii* transformed with pKU411	This study
pKU412/NR1491	*E. ludwigii* transformed with pKU412	This study
NR3901	Ceftazidime-resistant mutant of *E. ludwigii* NR1491, AmpD mutant (69delG)	This study
**Plasmids**		
pKU411	Wild type *ampR* (135Asp) of *E. cloacae* GN7471 cloned into pMW218	Kuga et al., 2000
pKU412	Mutant *ampR* (135Asn) of *E. cloacae* GN7471 cloned into pMW218	Kuga et al., 2000

The minimum inhibitory concentrations (MICs) of the various antimicrobial agents were determined for each strain by the agar dilution method according to CLSI guidelines (CLSI, 2018).

### Whole-Genome Sequencing and Analysis

The genomic DNA of NR1491 was prepared with a Qiagen Genomic-tip 500/G kit (Qiagen, Hilden, Germany) and subjected to whole-genome sequencing on the MiSeq X10 platform (Illumina, San Diego, CA, United States). Reads were trimmed in Trimmomatic and assembled to contigs with the SPAdes v. 3.8.1 genome assembler in caution mode (Bankevich et al., 2012).

Species were precisely identified based on their average nucleotide identity (ANI) and *in silico* DNA–DNA hybridization between strain NR1491 (GenBank accession no. BKZO00000000.1), the *E. cloacae* type strain ATCC 13047 (GenBank accession no. MTFV00000000.1), the *E. ludwigii* type strain EN-119 (GenBank accession no. JTLO00000000.1), and the *E. ludwigii* type strain AOUC-8/14 (GenBank accession no. LGIV00000000.1). Earlier studies recommended ANI of ∼95–96% as a species demarcation cutoff (Goris et al., 2007; Chun and Rainey, 2014).

Antimicrobial resistance genes were identified in the genome sequence with the ResFinder database^1^ using thresholds of 90% identity and 60% minimum length. β-lactamase genes including carbapenemases and extended-spectrum β-lactamases (ESBLs) were also assessed by PCR. PCR detection of carbapenemases (*bla*_IMP_, *bla*_VIM_, *bla*_KPC_, *bla*_OXA–48–like_, *bla*_NDM_, *bla*_GES_, *bla*_*IMI/NmcA*_, and *bla*_SME_) (Poirel et al., 2011; Hong et al., 2012; Nakano et al., 2018) and ESBLs (*bla*_TEM_, *bla*_SHV_, and *bla*_CTX–M_) (Dallenne et al., 2010) were performed as previously described. The sequence surrounding *bla*_NmcA_, a carbapenemase-encoding gene, was elucidated by PCR and Sanger sequencing to close the gaps between the contigs. Sequence alignment and analysis were performed with BLAST^2^ at NCBI (National Centre for Biotechnology Information, Bethesda, MD, United States). Multilocus sequence typing (MLST) of the *E. cloacae* isolates was performed as previously described (Miyoshi-Akiyama et al., 2013). Sequence types were assigned at the PubMLST database.^3^ The presence of mobile genetic elements was investigated using the MobileElementFinder (Johansson et al., 2021) and INTEGRALL (Moura et al., 2009). The plasmid content was assessed using PlasmidFinder (Carattoli et al., 2014).

### Selection of Third-Generation Cephalosporin-Resistant Mutants and Detection of Sequence Alterations

Third-generation cephalosporin-resistant mutants were obtained by plating ∼10^9^ CFU mL^–l^ late-logarithmic-phase NR1491 grown in Luria-Bertani (LB) broth and on LB agar plates containing ceftazidime or cefotaxime at 2×, 4×, 8×, 16×, and 32× MIC. The mutation frequencies were determined by dividing the colony density in CFU mL^–l^ on LB agar plates containing the antibiotic agents by the total colony density in CFU mL^–l^.

The DNA sequences of the selected mutants were determined by Sanger sequencing of *nmcR*, *ampR*, and *ampD* amplicons. The primers used are listed in Supplementary Table 1 (Radice et al., 2004). The nucleotides and amino acids of the selected mutants were compared with those of *E*. *ludwigii* NR1491 and EN-119.

### β-Lactamase Induction Assays

β-lactamase activity was measured in terms of the protein content in the extract and compared among cultures in 50 mM phosphate buffer (pH 7.0) at 30°C by spectrophotometry as previously described (Nakano et al., 2004). The protein concentrations were determined by the Bradford assay (Bradford, 1976). One unit of β-lactamase activity was defined as the amount of β-lactamase hydrolyzing 1 μmol cephalothin in 1 min at 30°C. The β-lactamase induction assays were performed by subjecting mid-logarithmic phase bacteria in Mueller-Hinton broth to β-lactams at 1/16×, 1/8×, 1/4×, 1/2×, and 1× MICs for 2 h (Kuga et al., 2000). The antibiotics cefpodoxime, clavulanic acid, cefoxitin, imipenem, and meropenem were used as inducers. The induction ratios were calculated in terms of the ratio of β-lactamase activity mg^–1^ protein in induced cells to the β-lactamase activity per mg^–1^ protein in uninduced cells.

### Measurement of *ampC* and *nmcA* mRNA Levels by qRT-PCR

The mRNA expression levels of *ampC* and *nmcA* with and without induction were determined by qRT-PCR as previously described (Nakano et al., 2017). Total RNA was extracted with the RNeasy protect bacteria mini kit and the RNase-free DNase set (Qiagen, Hilden, Germany) according to the manufacturer’s instructions. The qRT-PCR was performed in a StepOnePlus real-time PCR system (Applied Biosystems, Foster City, CA, United States) with a Power SYBR Green RNA-to-CT 1-Step kit (Thermo Fisher Scientific, Waltham, MA, United States) and 100 ng total RNA in a 20-μL reaction, according to the manufacturer’s instructions. The primers used are listed in Supplementary Table 1 (Doumith et al., 2009). The relative gene expression levels were calculated by the 2^–△△CT^ method. The mRNA of the housekeeping gene *rpoB* was chosen as the endogenous reference for relative quantification. The results are presented as the mRNA expression level compared with that of NR1491. The experiment was performed in triplicate. The final relative expression levels of *ampC* and *nmcA* were determined by calculating the averages for their transcripts. The coefficient of variation (SD/mean) among experiments was < 10%.

### Nucleotide Sequence Accession Numbers

The nucleotide sequences of the genetic regions surrounding *bla*_NmcA_ and the whole-genome DNA sequences of NR1491 were deposited in the GenBank database under accession numbers LC482123 and BKZO00000000.1, respectively.

## Results

### Identification of *bla*_NmcA_-Harboring *Enterobacter ludwigii* NR1491

The draft NR1491 genome (GenBank accession no. BKZO00000000.1) was obtained by MiSeq (Illumina, Sa, Diego, CA, United States), and average nucleotide identity (ANI) analysis using *E. cloacae* strain ATCC13047, *E. ludwigii* type strain EN-119, and *E. ludwigii* AOUC-8/14 as reference genomes. Their respective ANI values were 87.82, 98.96, and 98.97%.^4^ NR1491 was identified as *E. ludwigii* belonging to ST258.

### Antimicrobial Susceptibility and Resistance Genes

Antimicrobial susceptibility assays showed that *E. ludwigii* NR1491 was highly resistant to cephalothin, cefmetazole, carbapenems, and fosfomycin (> 512 μg mL^–l^) but susceptible to 3GC, piperacillin–tazobactam, cefepime, aztreonam, levofloxacin (≤0.06 μg mL^–l^), and gentamicin (0.5 μg mL^–l^) (Table 2). However, the MIC of cefotaxime increased when the agent was combined with clavulanic acid.

**TABLE 2:** Susceptibility and β-lactamase activity of *E. ludwigii* NR1491 and the mutant strains.

Strain	Gene on plasmid	AmpD characteristic	MICs (μg mL^–l^)^a^												β-Lactamase activity of (Unit mg^–l^ of protein)^c^		Fold increase in activity		Expression^e^			
																			Basal		Induced*^d^*	
			PIP	PIP/TAZ^b^	CEF	CPD	CTX	CTX/CLA^b^	CAZ	FPM	CFX	AZT	IPM	MER	Basal	Induced^d^	Induced/basal	Mutant/WT	*ampC*	*nmcA*	*ampC*	*nmcA*
NR1491	–	Wild type	4	2	>512	4	0.25	4	1	0.125	512	1	64	16	0.03	2.97	97.5	–	1	1	14.7	14.6
NR1491 (pKU411)	Wild type AmpR (135D)	Wild type	4	2	>512	2	0.25	2	1	0.125	512	1	64	16	0.03	2.94	101.1	1	0.7	1.1	14.6	15.3
NR1491 (pKU412)	Mutant AmpR (135N)	Wild type	32	8	>512	512	32	16	64	0.25	>512	32	32	16	3.06	4.54	1.5	100.3	96.4	1.0	205.6	2.3
NR3901	–	Mutant (69delG)	128	64	>512	256	32	16	64	0.5	>512	256	32	16	21.54	21.48	1.0	706.5	698.5	695.0	680.4	679.4

*^a^Antibiotics: PIP, piperacillin; TAZ, tazobactam; CEF, cephalothin; CPD, cefpodoxime; CTX, cefotaxime; CLA, clavulanic acid; CAZ, ceftazidime; FEP, cefepime; CFX, cefoxitin; AZT, aztreonam; IPM, imipenem; MER, meropenem.*

*^b^MICs were determined in the presence of tazobactam (4 μg mL^–l^) or clavulanic acid (4 μg mL^–l^).*

*^c^β-lactamase activities are the geometric means for three independent cultures. The standard deviations were within 10%.*

*^d^Induction was carried out with 32 μg mL^–l^ cefoxitin.*

*^e^Relative to the expression of NR1491 which was assigned a value of 1. Standard deviations were within 10%.*

Whole-genome analysis with ResFinder revealed the following resistance-encoding genes: *bla*_NmcA_ (carbapenemase), ACT-12 (AmpC β-lactamase), and *fosA2* (glutathione transferase; fosfomycin resistance). It also disclosed that the regulator genes *nmcR* and *ampR* were upstream of *bla*_NmcA_ and *ampC*, respectively. The entire nucleotide sequences of *bla*_NmcA_ and *nmcR* and the intercistronic region were determined by Sanger sequencing. The sequences were identical to that of *E*. *cloacae* NOR-1 (accession no. Z21956). PCR demonstrated that no other acquired β-lactamase gene was harbored. The regulatory gene sequences of *bla*_NmcA_ (*nmcR*, *ampR*, and *ampD*) were compared with that of the reference strains of ECC (NOR-1, EN-119, and AOUC-8/14); there are no mutations in these genes. Whole-genome analysis indicated that the insertion sequence (IS) elements and an integron were not encoded on the chromosome; the strain did not harbor a plasmid.

### Genetic Environment Analysis of *bla*_NmcA_

**FIGURE 1:**
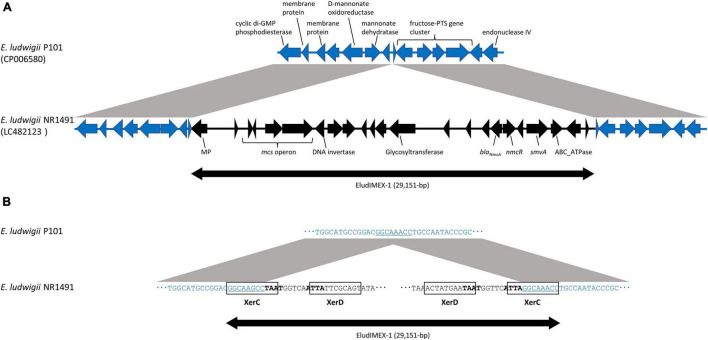
Schematic representations and nucleotide sequences of the genetic elements surrounding EludIMEX-1 of *E. ludwigii* NR1491 (GenBank accession no. LC482123) and the corresponding region of *E. ludwigii* P101 (GenBank accession no. CP006580). **(A)** Schematic representation of the genetic region of *E. ludwigii* NR1491 (black arrow) and *E. ludwigii* P101 (blue arrow). The gray regions between the NR1491 and P101 indicate > 99% nucleotide sequence identity. Insertion of the EludIMEX-1 element (double-headed arrow) in *E. ludwigii* NR1491 was observed. **(B)** Nucleotide sequences at the junctions of EludIMEX-1 (a part of left and right of the junctions) of *E. ludwigii* NR1491 and the corresponding region of *E. ludwigii* P101. The XerC/XerD binding sites are boxed, the conserved regions are boldfaced, and the consensus repeat sequences in XerC binding site of *E. ludwigii* NR1491 and corresponding sequences of *E. ludwigii* P101 are indicated using underlined letters.

The genetic environment of *bla*_NmcA_ was determined to be a 48,089-bp nucleotide fragment characterized by whole-genome and Sanger sequencing and deposited into GenBank under accession no. LC482123. A BLASTn analysis showed that the fragment was highly similar to *E. ludwigii* AOUC-8/14 (accession no. KR919803) (44,766/44,874 nucleotides; 99.76% identity). The *bla*_NmcA_ was located on a novel putatively mobile 29-kb element designated EludIMEX-1 inserted into the same chromosome location as that in *E. ludwigii* AOUC-8/14. Two imperfect 29-bp inverted repeat XerC/XerD binding sites associated with EludIMEX were identified at the chromosome–EludIMEX-1 junctions. The genetic regions were compared with the corresponding regions of *E. ludwigii* P101 (GenBank accession no. CP006580); the schematic representations are depicted in Figure 1. There were highly homologous regions (> 99% nucleotide sequence identity) and the insertion of EludIMEX-1. *bla*_NmcA_ was putatively integrated into the *E. ludwigii* chromosome via a Xer-dependent recombination mechanism mediated by EludIMEX-1, as described previously.

BLASTn analysis of NR1491 indicated that there were 10 strains of NmcA-positive ECC with > 99% nucleotide sequence identities (Supplementary Table 2). The query coverage includes the gene regions of the EludIMEX-1 element and the surrounding regions with consensus repeat sequences and a binding site. The EludIMEX-1 was integrated into the chromosome at the same site, in these strains. The genotypes of these strains were including ST258 (*n* = 2) same with NR1491, ST282 (*n* = 2), ST257, ST260, ST374, ST714 (AOUC-8/14), ST748, and ST1724.

### Antibiotic Inducibility of β-Lactamase

**FIGURE 2:**
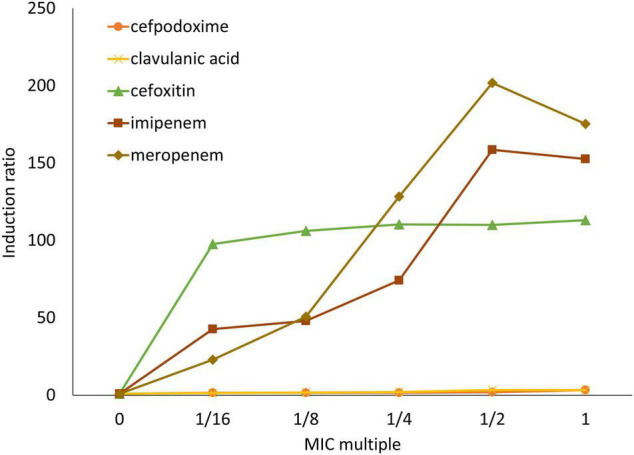
Induction ratios of β-lactamase activity in response to antibiotics on *E. ludwigii* NR1491. β-lactamase inducers were cefpodoxime (○), clavulanic acid (×), cefoxitin (△), imipenem (□), and meropenem (◇).

The antibiotic inducibility of β-lactamase was analyzed in NR1491 (Figure 2). Cefpodoxime and clavulanic acid were slightly inducer in a concentration-dependent manner. They yielded only a maximum 3.3 × induction of the MIC. Conversely, cefoxitin, imipenem, and meropenem were strong β-lactamase inducers. The carbapenems imipenem and meropenem induced β-lactamase in a concentration-dependent manner to 159× and 202×, respectively, at half their MIC. In contrast, the cefoxitin induction rate was concentration-independent and remained virtually unchanged (98–113 ×) across the tested concentrations (1/16–1 × MIC).

### Properties of the Selected Third Generation Cephalosporins-Resistant Mutants

The 3GC-resistant mutants were selected with cefotaxime and ceftazidime at 2×, 4×, 8×, 16×, and 32× MIC. The antibiotic-resistant mutants were consistently obtained at a mutation frequency of ∼10^–6^–10^–7^ both for cefotaxime and ceftazidime (Table 3).

**TABLE 3:** Amino acid and nucleotide changes in AmpD of ceftazidime- or cefotaxime-resistant mutants of *E. ludwigii* NR1491 and their antimicrobial susceptibilities.

Mutation	Selective agents (μg mL^–l^)^a^	No. of selected strains	Amino acid and nucleotide changes detected in AmpD (no. of strains)^b^	MIC range (μg mL^–l^)^a^										
				PIP	PIP /TAZ	CPD	CTX	CTX /CLA	CAZ	FPM	CFX	AZT	IPM	MER
Missense	CTX (1, 2, 4, 8, 16) CAZ (2, 4, 8, 16, 32)	36	M1I (2), N35K (2), L56P, L56Q, T55P, H75Y, I78N (8), I78S, G82V, W95G, G98D, S100L (4), L117R, E118G, T123P (7), T137P, G166A, D170Y	16–128	8–32	128–256	8–32	8–16	16–64	0.125–0.5	512–> 512	32–256	16–64	8–16
Non-sense	CTX (1, 8, 16) CAZ (8, 32)	7	W7*, E26*, E83*, Q86*, Y102*(2), Q103*	128	32	256–512	16	8–16	64	0.25–0.5	512	256	16–32	8–16
Frameshift	CTX (8, 16) CAZ (4, 16, 32)	5	69delG, 129_130insT, 270_271insT, 372delC, 401_404del	128	16–32	256–512	16–32	8–16	64	0.5	512–> 512	256	32	16

*^a^Antibiotics: PIP, piperacillin; TAZ, tazobactam; CEF, cephalothin; CPD, cefpodoxime; CTX, cefotaxime; CLA, clavulanic acid; CAZ, ceftazidime; FEP, cefepime; CFX, cefoxitin; AZT, aztreonam; IPM, imipenem; MER, meropenem.*

^b^Nucleotide and deduced amino acid differences in AmpD were compared with E. ludwigii NR1491 and EN-119. *, stop codon; del, deletion; ins, insertion.

To investigate the molecular mechanism of 3GC resistance in these mutants, 48 clones were randomly selected from each condition. The regulator genes *ampR, nmcR*, and *ampD* were sequenced and compared to those of the parent *E. ludwigii* NR1491 and wild type EN-119 strains. Sequence data revealed that only *ampD* was altered in all cases whereas neither *ampR* nor *nmcR* was changed. Of the 48 3GC-resistant mutants, 34 had possible loss-of-function caused by missense mutations including 18 amino acid substitutions at 16 positions in *ampD* (Table 3). Premature termination of the AmpD protein was found in 14 mutants. Seven had nonsense mutation at six positions, five had frameshift mutations (three deletions and two insertions), and two had missense mutations in which the start codon (ATG) was changed to Ile (ATA) at position 1. Its effect was transcriptional decay. These mutations were scattered throughout the entire *ampD* sequence. Moreover, the nucleotide substitutions and mutation types and locations did not differ among selective agents and concentrations. However, certain mutants had the same missense mutation positions. Eight were I78N, seven were T123P, and four were S100L. These mutation positions may have been influential to AmpD activity. These mutants were resistant to 3GC presumably as a consequence of loss of AmpD function via the introduction of substitution mutations or decay of the transcript containing the premature stop codon.

For the mutants, the MICs were determined for the selected β-lactams (Table 3). Compared with the MICs for the parent strain NR1491, the MICs of 3GC (cefotaxime and ceftazidime) and aztreonam for the mutants had increased by ≥ 16 ×. Thus, these strains were reclassified from susceptible to resistant. The MICs of cefepime increased by only 1–4 × relative to NR1491 and were beyond the clinical resistance breakpoint. In contrast, the MICs of the carbapenems were almost always the same as those for NR1491.

### Effects of Regulator Genes on β-Lactamase Expression

To evaluate the effects of *ampR* and *ampD* on NR1491 drug susceptibility, β-lactamase activity and mRNA expression of AmpC and NmcA with and without induction were analyzed in the *ampR* mutant NR1491 (pKU412) and the *ampD* mutant NR3901 (Table 2). Induction was carried out with 32 μg mL^–l^ cefoxitin. The β-lactamase activity of NR1491 increased by ∼100 × and AmpC and NmcA were upregulated by ∼14.7× and 14.6×, respectively, in response to induction. *ampC* and *nmcA* expression levels were equally influenced by the induction.

NR3901 bore an *ampD* mutant (69delG) and was isolated by selection with 32 μg mL^–l^ ceftazidime. The MICs of 3GC and aztreonam increased by ≥ 64 × and they were reclassified as highly resistant. The β-lactamase activity of NR3901 increased by ∼706.5 and ∼704.5 × with and without induction, respectively, compared with the NR1491 basal condition. The *ampC* and *nmcA* expression levels in NR3901 both increased by ∼700 × in the presence and absence of induction. The *ampC* and *nmcA* expression levels in NR1491 both increased by ∼15 × in response to induction. Hence, *ampD* equally induced *ampC* and *nmcA* expression. NR3901 was highly drug-resistant because it acquired the *ampD* mutation which derepressed *ampC* and *nmcA* expression.

NR1491 (pKU412) was obtained by transfecting the *ampR* mutant (135Asn) clone plasmid pKU412 into NR1491. The MICs of NR1491 (pKU412) were elevated as they were for NR3901. The β-lactamase activity had increased by ∼100 × at basal condition. *ampC* expression also increased by ∼100 × whereas that of *nmcA* did not change. AmpR may induce AmpC β-lactamase but does not affect *nmcA* expression. NR1491 (pKU412) induction resulted in a 1.5 × increase which suggests partial derepression. *ampC* expression increased by ∼200 × after induction. However, *nmcA* expression only doubled despite NR1491 expression increasing by ∼15 ×. Plasmid pKU411 comprising the wild type *ampR* (135Asp)-harboring strain NR1491 (pKU411) was compared with the *ampR* mutant strain and used to verify it. The MICs and β-lactamase activity of NR1491 (pKU411) were nearly the same as those for NR1491.

## Discussion

The incidence of CPE is increasing globally. However, it has seldom (0.34%) been detected in Japan (Ohno et al., 2017). The most common carbapenemase genotype detected in Japan is IMP. Here, we isolated NmcA carbapenemase-producing ECC. NmcA carbapenemase has occasionally been reported for ECC in Europe, North America, and South America (Radice et al., 2004; Antonelli et al., 2015; Boyd et al., 2017). To the best of our knowledge, this is the first reported clinical isolation of an NmcA carbapenemase producer in Japan.

ANI revealed that NR1491 was, in fact, *E. ludwigii* belonging to ST258. A previous study reported that *bla*_NmcA_ was highly associated with *E. ludwigii.* ST258 is a genotype of the NmcA carbapenemase producer (Boyd et al., 2017). The genetic environment of *bla*_NmcA_ was nearly identical to that of *E. ludwigii* AOUC-8/14. Thus, *bla*_NmcA_ was putatively integrated into the chromosome by EludIMEX-1 via a Xer-dependent recombination mechanism as previously described for *E. ludwigii* AOUC-8/14 (Antonelli et al., 2015). Interestingly, *E. ludwigii* AOUC-8/14 was isolated from a Japanese tourist in Italy. These strains may have been concealed in Japan and unintentionally isolated in the present study. Comparative genome analysis revealed that there were 10 strains including AOUC-8/14, which have high homology regions with NR1491. These EludIMEX-1 was integrated in the chromosome at the same site as in NR1491. The genotypes of these strains were different; the EludIMEX-1 insertion event has possibility occurred in these STs strains.

As with *bla*_NmcA_, NR1491 coexists with the regulator gene *nmcR*. A β-lactamase induction assay on NR1491 showed that it was weakly induced by clavulanic acid which was already known to be an inhibitor of Class A β-lactamases. Thus, it is inhibitory against NmcA β-lactamase (Mariotte-Boyer et al., 1996). On the other hand, clavulanic acid also induces chromosomally mediated AmpC β-lactamases in several Enterobacteriaceae (Drawz and Bonomo, 2010). Here, clavulanic acid induced β-lactamases via transcriptional regulator genes and not by inhibiting NmcA. The MIC of cefotaxime was increased in combination with clavulanic acid while imipenem and meropenem induced β-lactamase in a concentration-dependent manner. Previous study of its kinetic parameters show that NmcA demonstrated unusually strong hydrolytic activity toward imipenem and meropenem (Mariotte-Boyer et al., 1996). Therefore, NmcA producers are highly resistant to carbapenems as their inducers are upregulated and they are potently hydrolytic to carbapenems. Cefoxitin is a strong, stable, dose-independent β-lactamase inducer (100 × induction). The catalytic efficiency (*k*_cat_/*K*_m_) of cefoxitin is lower than those of the carbapenems but its MIC is comparatively higher (Mariotte-Boyer et al., 1996) possibly because of its high and stable inducibility.

To elucidate the mechanism of β-lactamase induction in NR1491, 3GC-resistant mutants were selected with cefotaxime and ceftazidime. The mutation frequencies were 10^–6^–10^–7^ as previously described (Naas et al., 2001). Forty-eight randomly selected clones had variable susceptibilities to 3GC, piperacillin–tazobactam, and aztreonam (Table 3). A DNA sequence analysis revealed that all mutants presented with nucleotide substitutions (frameshift, missense, or nonsense) in *ampD* alone. In contrast, *ampR* and *nmcR* were unchanged. The mutants were resistant to 3GC and probably had loss of AmpD function. Premature AmpD termination with a stop codon or frameshift induced strong 3GC resistance. The MICs of the *ampD* mutant strains with missense mutation had different 3GC resistance levels. The degree of 3GC resistance depended on the position of the substitution at the core residues of the active site of AmpD.

In the present study, no mutants of the transcriptional regulator genes *ampR* and *nmcR* were obtained. We investigated the effects of *ampR* in the presence of a mutant or wild type *ampR* clone plasmid. AmpR is a member of the lysR family and regulates the expression of chromosomal AmpC β-lactamase. Nevertheless, *ampR* mutants cause constitutive AmpC overproduction (75–470× increase) irrespective of induction (Kuga et al., 2000). In the enzyme activity assay, NR1491 (pKU412) increased β-lactamase activity by 100 × at the basal level compared with NR1491, also upregulating *ampC*. β-lactamase activity in the NR1491 strain with wild type *ampR* was increased by 100 × by induction and *ampC* and *nmcA* were each upregulated 15×. Whereas NR1491 with wild type *ampR* upregulated *nmcA* 15×, *nmcA* expression in NR1491 (pKU412) only doubled. Mutant *ampR* may negatively regulate *nmcA* expression. Putative *ampR* binding sequences in the *E. cloacae ampR–ampC* intergenic region were highly conserved with *nmcR–bla*_NmcA_ and cross-reaction may have occurred (Naas and Nordmann, 1994). Earlier studies suggested that *ampR* is a global transcriptional regulator affecting the expression of several genes as well as *ampC* (Balasubramanian et al., 2012). NmcR was described as a positive regulator both in the absence and especially in the presence of a β-lactam inducer. In the absence of inducer, *ampR* is a negative regulator of *ampC* expression. In its presence, it positively regulates *ampC* expression (Naas and Nordmann, 1994). These findings suggest that even with available induction, mutant *ampR* represses the expression of *nmcA*. We believe this is the first study to describe the association between *ampR* and *nmcA* expression.

NmcR mutant strain has not been identified yet; therefore, the effect of *nmcR* mutations on the expression of *nmcA* could not be assessed. Point mutations in *nmcR* may be required to enhance its efficacy as an activator of *nmcA* in the same way as mutant *ampR* (such as that with a change in Asp135Asn). Alternatively, it may repress *nmcA* expression in the same way as wild type *ampR*. In a previous study, *ampR* mutants were obtained from the *ampD* mutant strain at a very low frequency (Kuga et al., 2000). The *ampD* mutant strain NR3901 selected *nmcR* mutants using ceftazidime at double and quadruple the MIC (128 and 256 μg mL^–l^). Nevertheless, no *nmcR* mutants were obtained (data not shown). *nmcR* mutants may be selected using *ampD*-mutant *E. coli* strains carrying *bla*_NmcA_ and *nmcR* cloning plasmids. NR1491 co-harbors *ampC* on the chromosome. The observed differences in β-lactamase substrate specificity may influence selection conditions, and further investigation is needed to clarify whether *nmcR* mutation increases resistance by upregulating *nmcA*. This study has certain limitations. The analysis included only one strain and the conclusions were based on the results from this strain. Therefore, further studies are required to clarify the mechanisms of *nmcR* by selecting the *nmcR* mutant strains using other NmcA-producing ECC or IMI (closely related carbapenemase NmcA) producing *E. coli*. Moreover, the *ampR* mutation in NR1491 resulted in strong 3GC resistance via *ampC* overexpression. However, it remains unclear as to how mutant *ampR* stimulates *nmcA* transcription through induction. Future research should aim to elucidate the function of *ampR*.

NR3901 with an *ampD* mutation presented with a 700 × increase in β-lactamase activity as well as upregulated *ampC* and *nmcA*. Consequently, the MICs of 3GC were elevated. It indicates that *ampD* mutation has a similar influence on the expression of both *ampC* and *nmcA*, suggesting that structurally unrelated genes could be under the control of an identical regulatory system (Naas et al., 2001). The MICs for NR3901 and NR1491 (pKU412) bearing the *ampR* mutant plasmid were nearly equal. The upregulated *nmcA* in NR3901 had no effect on MIC compared with NR1491 (pKU412). Moreover, the MICs of the carbapenems for NR3901 were almost always the same as those of the parent strain despite the *ampD* mutant constitutively upregulating *ampC* and *nmcA*. High-level *nmcA* expression may elevate the MICs of carbapenems; in fact, the MICs were same as those of the parent strain. NmcA metabolism and its associated physiology may be connected with MICs. However, further experimentation is required to clarify this mechanism.

Here, we detected the NmcA-producing strain NR1491 in a hospital patient. Examination of MIC patterns showed high resistance to carbapenems but susceptibility to 3GC. The *ampD* mutant strains were identified among clinical isolates of ceftazidime-resistant *E*. *cloacae* as previously reported (Kaneko et al., 2005). Therefore, *ampD*-mutant NmcA producers may occur and interfere with the clinical detection of their wild type counterparts. We characterized NmcA producers that were highly resistant to carbapenems and yet susceptible to cefepime, whether they acquired the *ampD* mutation. In future works, it would be informative to compare these strains with the Big Five carbapenemases (KPC, IMP, NDM, VIM, and OXA).

## Conclusion

In the present study, we identified the *E. ludwigii* isolate NR1491 in Japan that produces NmcA. The *bla*_NmcA_ was located on a novel 29-kb putatively mobile element designated EludIMEX-1 identical in structure to that previously described in Europe. Induction studies revealed that the *ampD* mutants equally upregulated β-lactamases *nmcA* and *ampC* and were highly resistant to 3GC. However, the observed increase in the MIC value of 3GC was caused mainly by *ampC* overexpression. The *ampR* mutants also upregulated *ampC*, however, that of *nmcA* seemed to be repressed. Further research is necessary to elucidate the functions of *ampR* and *nmcR*.

## Data Availability Statement

The datasets presented in this study can be found in online repositories. The names of the repository/repositories and accession number(s) can be found in the article/Supplementary Material.

## Author Contributions

RN conceived and designed the experiments, performed the experiments, analyzed and interpreted the data, and wrote the manuscript. YY contributed reagents and materials and performed the experiments. AN, YS, KS, RS, MO, and KN performed the experiments, validation, and interpreted the data. AK contributed reagents and materials, conceived, and designed the experiments. AS and HY supervision and project administration. All authors contributed to manuscript, read, and approved the submitted version.

## Conflict of Interest

RS and MO were employed by the company BML Inc. The remaining authors declare that the research was conducted in the absence of any commercial or financial relationships that could be construed as a potential conflict of interest.

## Publisher’s Note

All claims expressed in this article are solely those of the authors and do not necessarily represent those of their affiliated organizations, or those of the publisher, the editors and the reviewers. Any product that may be evaluated in this article, or claim that may be made by its manufacturer, is not guaranteed or endorsed by the publisher.

## Footnotes

1
https://cge.cbs.dtu.dk/services/ResFinder/

2
https://blast.ncbi.nlm.nih.gov/Blast.cgi

3
http://pubmlst.org/ecloacae/

4
https://www.ezbiocloud.net/tools/ani
