# Supplementary material for: The Role of nmcR, ampR, and ampD in the Regulation of the Class A Carbapenemase NmcA in Enterobacter ludwigii
Source: Front Microbiol. 2022 Jan 12;12:794134. doi: 10.3389/fmicb.2021.794134 (PMC8790168; doi:10.3389/fmicb.2021.794134)
Supplement: Supplementary file 1 [file Table_1.docx]

**Supplementary Table S1.** Primers used in the present study.

| **Gene** | **Sequence 5′→3′** | **Purpose** | **Reference** |
| --- | --- | --- | --- |
| *nmcR*f | GCATTGATATACCTTTAGCAGAGA | Sequencing | Radice et al., 2004 |
| *nmcR*r | CGGTGATAAAATCACACTGAGCATA | Sequencing | Radice et al., 2004 |
| *ampR*f | ATGACACGCAGCTATTTACCGC | Sequencing | This study |
| *ampR*r | AGCCACTGCGCAAACTCACG | Sequencing | This study |
| *ampD*f | AGGCGGCATGATAAAACTCC | Sequencing | This study |
| *ampD*r | TCATGTTATCTCCTTATCTG | Sequencing | This study |
| *ampC*f | GGGCGATCCGGTGACCAAATACT | Expression analysis | This study |
| *ampC*r | ACCGCCAGAGCCCCAAACAGACC | Expression analysis | This study |
| *nmcA*f | ATATGGCTGCTGCTGCTTTACAAT | Expression analysis | This study |
| *nmcA*r | CACGCTCATCGCCTGGAATAG | Expression analysis | This study |
| *rpoB*f | AAGGCGAATCCAGCTTGTTCAGC | Expression analysis | Doumith et al., 2009 |
| *rpoB*r | TGACGTTGCATGTTCGCACCCATCA | Expression analysis | Doumith et al., 2009 |

**Supplementary Table S2.** Sequence type, identities, and accession numbers of the *Enterobacter cloacae* complex isolates which has higher homology region including EcloIMEX-1 and the surrounding region of NR1491

| Strains | ST | Identities (%) | Accession number |
| --- | --- | --- | --- |
| NR1491 | 258 | 48089/48089 (100) | LC482123 |
| FDA-CDC-AR_0132 | 282 | 47977/48089 (99.77) | NZ_CP027618 |
| FDA-CDC-AR_0164 | 1724 | 47964/48089 (99.74) | NZ_CP028950 |
| AOUC-8/14 | 714 | 44,766/44,874 (99.75) | KR919803 |
| N11-1141 | 258 | 31312/31313 (99.99) | KR057493 |
| N12-1563 | 258 | 31309/31313 (99.99) | KR057496 |
| N13-1362 | 282 | 31305/31313 (99.97) | KU870976 |
| N10-3276 | 257 | 31305/31313 (99.97) | KR057492 |
| N14-1767 | 374 | 31297/31313 (99.95) | KU870979 |
| N15-2827 | 748 | 31294/31313 (99.94) | KU870985 |
| N12-1562 | 260 | 31072/31311 (99.24) | KR057495 |
